# Ecological Diversity of Migratory Birds and Their Associated Bacterial Species in South Korea: A Preliminary Study Including Antimicrobial Resistance Profiles

**DOI:** 10.3390/vetsci12121157

**Published:** 2025-12-04

**Authors:** Hyungju Lim, Jun-Gyu Park, Chung-Do Lee, Gun Lee, Jaewoo Choi, Hyeon Jeong Moon, Woo-Yuel Kim, Seulgi Seo, Gi-Chang Bing, Bock-Gie Jung, Yeong-Bin Baek, Dae Sung Yoo, Jun Bong Lee, Kwang-Jun Lee, Sang-Ik Park

**Affiliations:** 1 Jeollanamdo Veterinary Service Laboratory, Gangjin 59213, Republic of Korea; 2 Department of Veterinary Zoonotic Diseases, College of Veterinary Medicine, Chonnam National University, Gwangju 61186, Republic of Korea; 3 BK21 FOUR Program, Department of Veterinary Pathology, College of Veterinary Medicine, Chonnam National University, Gwangju 61186, Republic of Korea; 4 Department of Veterinary Pathology, College of Veterinary Medicine, Chonnam National University, Gwangju 61186, Republic of Korea; 5 The Wings Co., Ltd. 807, Industry-Academic Cooperation Center 1, Chonnam National University, Gwangju 61186, Republic of Korea; 6 Human & Nature Institute (HNI), 115-1704, Sejong-si 30124, Republic of Korea; 7 Department of Veterinary Microbiology, College of Veterinary Medicine, Chonnam National University, Gwangju 61186, Republic of Korea; 8 Department of Veterinary Epidemiology, College of Veterinary Medicine, Chonnam National University, Gwangju 61186, Republic of Korea; 9 Department of Food and Environmental Hygiene, College of Veterinary Medicine, Chonnam National University, Gwangju 61186, Republic of Korea; 10 Division of Zoonotic and Vector-Borne Disease Research, Center for Infectious Diseases Research, National Institute of Health, Cheongju 28159, Republic of Korea

**Keywords:** migratory birds, bacteria, antimicrobial resistance, South Korea

## Abstract

Migratory birds travel long distances and interact with diverse environments, making them potential reservoirs and disseminators of antimicrobial-resistant bacteria. This study investigated the species distribution of migratory birds, bacterial isolates from bird internal organs, and the corresponding antimicrobial resistance (AMR) profiles in South Korea. A total of 35 bird carcasses representing 20 species were collected from five major stopover sites on the Sinan-gun islands along the East Asian–Australasian Flyway. More than half of the sampled birds belonged to the genus *Emberiza*, reflecting the prevalence of small migratory passerines in coastal habitats. From these carcasses, 54 bacterial isolates belonging to 24 species were identified, including *Enterococcus* spp., coliforms such as *Enterobacter* spp. and *Escherichia coli*, and opportunistic pathogens including *Bacillus* spp., *Staphylococcus* spp., and *Serratia* spp. Antimicrobial susceptibility testing revealed that 18 isolates (33.3%) were multidrug-resistant (MDR). *Enterococcus* isolates displayed high resistance to tigecycline and daptomycin, and two vancomycin-resistant strains were identified. Coliform isolates were resistant to third- and fourth-generation cephalosporins, carbapenems, and colistin. The two *E. coli* strains exhibited concurrent carbapenem–colistin resistance, posing a significant public health concern. These findings provide the first organ-level AMR dataset for migratory birds in South Korea and highlight the potential role of small passerines as ecological sentinels of environmental contamination. The detection of MDR strains underscores the need to integrate wildlife surveillance into One Health strategies for AMR monitoring.

## Simple Summary

Migratory birds travel long distances and encounter various environmental sources of bacteria. In this study, we examined 35 dead migratory birds from key stopover sites in South Korea. Most of these birds were from the *Emberiza* genus, which often uses coastal islands during migration. We identified 54 bacterial isolates from various organs of birds. The main types of bacteria include *Enterococcus*, coliform bacteria, *Bacillus*, *Staphylococcus*, and *Serratia*. Many of these bacteria are resistant to clinically important antimicrobials. One-third of them were resistant to multiple antimicrobials, including vancomycin-resistant *Enterococcus* and carbapenem- and colistin-resistant *Escherichia coli* strains. These results suggest that migratory birds may help spread antimicrobial-resistant bacteria and cause environmental contamination. Thus, it is important to continue monitoring wildlife to understand its role in public health.

## 1. Introduction

Migratory birds play a crucial epidemiological role in the spread of microorganisms due to their long-range movements and exposure to anthropogenic sources such as urban wastewater and landfills [1]. They acquire and disperse bacteria across regions by traversing long expanses of water, deserts, and forests during migrations [2]. Each year, approximately 5 billion birds traverse continents [3], facilitating the global dissemination of various bacterial species. Migratory birds have been identified as carriers of various opportunistic pathogens, including *Escherichia coli* [4], *Enterococcus* [5], *Salmonella* [6], *Campylobacter* [7], *Listeria monocytogenes* [8], and *Staphylococcus* [9]. Moreover, indirect transmission of these pathogens to humans has been reported [10].

In addition to their role as carriers of surface- and gut-associated pathogens, birds can harbor bacteria within internal organs, such as the liver, spleen, kidneys, lungs, and gastrointestinal tract. Necropsy-based surveys of wild and captive birds have shown that systemic infections with bacterial pathogens often lead to colonization of these organs, including *E. coli*, *Salmonella*, *L. monocytogenes*, *Pasteurella multocida*, *Erysipelothrix rhusiopathiae*, and other Gram-negative bacteria [8,11]. However, most microbiome studies on birds have focused on fecal or intestinal communities [12,13], and organ-specific, culture-based data for migratory birds is relatively scarce, particularly outside the context of recognized disease outbreaks. In South Korea, previous studies have mainly targeted specific enteric or respiratory pathogens, such as *Campylobacter* spp., *Salmonella* spp., *Chlamydia psittaci*, and avian influenza viruses, using cloacal swabs, feces, or pooled organ samples [14,15]. To our knowledge, few studies have systematically compared culturable bacterial communities across multiple internal organs of migratory birds along the East Asian–Australasian Flyway (EAAF).

South Korea is located in the EAAF, one of the nine major migratory routes used by more than 50 million birds during their migration for breeding and wintering [16,17]. The large areas along the southwestern coast of the Korean Peninsula provide important habitats for migratory birds [18]. These coastal wetlands serve as crucial stopover sites where birds can rest and refuel during spring and autumn. Each year, approximately 50 bird species visit South Korea and interact with wetlands, agricultural landscapes, and urban environments [19]. Among these, buntings of the genus *Emberiza* are the most common migratory songbirds in South Korea. Long-term analyses have shown that species such as the Yellow-throated (*E. elegans*) and Black-faced (*E. spodocephala*) buntings remain common, playing a significant role in shaping the bunting community composition [20]. Despite a recent decline in its population, *E. elegans* remains a major bunting species in South Korea. It breeds in the Russian Far East, northern China, and the Korean Peninsula and winters in China, Japan, and Taiwan [21]. Although research on the gut microbiota of *Emberiza* is limited, a previous study identified the dominant bacterial phyla in the microbiota of *E. jankowskii* as Proteobacteria (52.45%), Firmicutes (13.87%), Bacteroidetes (5.76%), and Actinobacteria (4.95%) [22].

In particular, island archipelagos in Jeollanam-do such as Sinan-gun provide extensive tidal flats and coastal agricultural mosaics that have been recognized as internationally important stopover and wintering habitats within the EAAF [18,23,24]. Seasonal surveys in the Sinan-gun islands have documented high species richness and pronounced turnover in migratory bird communities throughout spring, summer, autumn, and winter, underscoring the ecological connectivity between these coastal landscapes and distant breeding and wintering areas [23]. These island complexes support large populations of migratory shorebirds and waterbirds, such as Kentish Plover (*Charadrius alexandrines*) and Little Egret (*Egretta alba modesta*), as well as abundant migratory passerines including Barn Swallow (*Hirundo rustica*) and Eurasian Tree Sparrow (*Passer montanus*) [25]. Moreover, an overwhelming proportion of the Korean avifauna—up to ~90% of bird species—is migratory, highlighting the national importance of conserving these stopover sites [26]. Therefore, carcass sampling along the southwestern coast, including island habitats used by *Emberiza* buntings and other small passerines, provides an opportunity to link organ-level bacterial carriage with well-characterized patterns of migratory bird movement and habitat use in this region.

Antimicrobial resistance (AMR) has become a significant threat to public health in the 21st century. The overuse and misuse of antimicrobials in human medicine and livestock farming has led to the spread of antimicrobial-resistant bacteria [27]. Although considerable attention has been given to monitoring AMR in clinical- and livestock-associated bacterial isolates, there has been relatively less focus on tracking AMR in wild animals [28]. However, wild animals, particularly migratory birds, are important for establishing ecological connections between humans, livestock, and the environment [29]. Therefore, gaining insight into the detection of antimicrobial-resistant bacteria in wild animals is essential for developing comprehensive surveillance systems within the One Health framework. Recent studies have demonstrated the presence of critically important antimicrobials (CIAs)-resistant bacteria in migratory birds. Notably, carbapenem-resistant and extended-spectrum β-lactamase (ESBL)-producing *E. coli* [30,31,32,33,34], vancomycin-resistant *Enterococcus* (VRE) [5,35,36,37], fluoroquinolone-resistant *Salmonella* [38,39] and *Campylobacter* [14,40], and methicillin-resistant *Staphylococcus* (MRSA) [37,41] have been isolated from birds worldwide. These findings highlight the global importance of migratory birds as potential reservoirs of clinically important bacteria.

Despite its geographic importance, comprehensive monitoring of AMR in migratory birds remains insufficient in South Korea. Therefore, this study aimed to investigate the species distribution and AMR profiles of bacterial isolates from migratory birds in South Korea. We hypothesized that birds in this region may harbor multidrug-resistant (MDR) bacteria, including those resistant to CIAs for human medicine, reflecting environmental contamination and potential interspecies transmission of AMR. By integrating ecological, microbiological, and AMR data, this study provides the first baseline evidence on the occurrence and diversity of antimicrobial-resistant bacteria in migratory birds in South Korea.

## 2. Materials and Methods

### 2.1. Sampling Methods

We selected five coastal areas in the Sinan-gun islands that are historically known as major habitats for migratory birds in South Korea. These areas were designated by the Korean Ministry of Environment as regular survey sites to monitor the migratory status of wild birds that use the Yellow Sea as a stopover site [18]. Sampling was conducted in vegetated areas near wetlands and bird-resting habitats at July and October in 2025. Thirty-five carcasses of birds found dead near stopover sites were collected for analysis. When necropsy could not be conducted on the sampling day, the carcasses were stored at −20 °C overnight to prevent further tissue decomposition. All carcasses were stored for the shortest possible duration, and tissues were processed immediately after thawing to limit additional stress on bacterial cells. In most cases, necropsy and bacterial isolation were performed on the same day as sample collection. During necropsy, major organs, including the liver, spleen, kidney, gastrointestinal tracts, bursa of Fabricius, lungs, brain, and trachea, were collected aseptically. All tissues were homogenized using beads (Bertin technologies, Montigny-le-Bretonneux, France). Bird species were identified by morphological characteristics confirmed by field ornithologists, and biosafety precautions were followed during carcass handling to ensure ethical and safe procedures.

### 2.2. Bacterial Isolation

For bacterial isolation, one loop of the organ homogenate was streaked onto blood agar plates (BAP) (Synergy Innovation, Gyeonggi, Korea). The plates were examined for visibly well-developed colonies after incubation at 37 °C for 24 h. Each colony was streaked onto a BAP to obtain pure colonies. The resulting colonies were stored at −80 °C in 25% (*v*/*v*) glycerol stocks. Each isolate was identified using a matrix-assisted laser desorption/ionization time-of-flight mass spectrometry (MALDI-TOF MS) system (Bruker Daltonics, Bremen, Germany) according to the manufacturer’s instructions. MALDI-TOF MS was validated to provide accuracy comparable to 16S rRNA gene sequencing [42,43].

### 2.3. Antimicrobial Susceptibility Test

The antimicrobial susceptibility of bacterial isolates was assessed by determining the minimum inhibitory concentrations (MICs) using the broth microdilution method with the Sensititre panels (TREK Diagnostic Systems, Cleveland, OH, USA). Briefly, bacterial colonies grown on BAP were suspended in 2 mL of distilled water to achieve a McFarland standard of 0.5. These bacterial suspensions were subsequently diluted with 11 mL of cation-adjusted Muller Hinton broth (TREK Diagnostic Systems, Cleveland, OH, USA) and dispensed onto a Sensititre panel. The panels were incubated at 37 °C for 20 h, and susceptibility was interpreted in accordance with the guidelines of the Clinical and Laboratory Standards Institute (CLSI, 2024), National Antimicrobial Resistance Monitoring System (NARMS, 2024), and European Committee on Antimicrobial Susceptibility Testing (EUCAST, 2024). However, for some bacterial species, breakpoints required to classify antimicrobial susceptibility (susceptible, intermediate, or resistant) are not available in any of the interpretive standards. To maintain methodological rigor and data transparency, these isolates were reported as “not interpretable” rather than being excluded or arbitrarily categorized.

For Gram-positive bacteria, 16 antimicrobials including tetracycline (TET, 2–128 μg/mL), ciprofloxacin (CIP, 0.25–16 μg/mL), daptomycin (DAP, 0.5–32 μg/mL), erythromycin (ERY, 1–64 μg/mL), tylosin (TYL, 1–64 μg/mL), linezolid (LZD, 0.5–16 μg/mL), tigecycline (TIG, 0.12–2 μg/mL), gentamicin (GEN, 128–2048 μg/mL), kanamycin (KAN, 128–2048 μg/mL), streptomycin (STR, 128–2048 μg/mL), quinupristin/dalfopristin (QUD, 1–32 μg/mL), vancomycin (VAN, 2–32 μg/mL), ampicillin (AMP, 1–16 μg/mL), chloramphenicol (CHL, 2–32 μg/mL), florfenicol (FLO, 2–32 μg/mL), and salinomycin (SAL, 2–32 μg/mL) were tested using the KRVP2F panel. For Gram-negative bacteria, 16 antimicrobials including CIP (0.12–16 μg/mL), amoxicillin/clavulanic acid (AMC, 2–32 μg/mL), cefoxitin (CXI, 1–32 μg/mL), CHL (2–64 μg/mL), STR (16–128 μg/mL), GEN (1–64 μg/mL), TET (2–128 μg/mL), nalidixic acid (NAL, 2–128 μg/mL), ceftazidime (CTZ, 1–16 μg/mL), trimethoprim/sulfamethoxazole (TRS, 0.12–4 μg/mL), cefepime (CEP, 0.25–16 μg/mL), cefotaxime (CTA, 0.5–8 μg/mL), meropenem (MER, 0.25–4 μg/mL), AMP (2–64 μg/mL), colistin (COL, 2–16 μg/mL), sulfisoxazole (SIS, 16–256 μg/mL) were tested using the KRNV6F panel.

## 3. Results

### 3.1. Species Distribution of Migratory Birds and Bacterial Isolates

**Figure 1:**
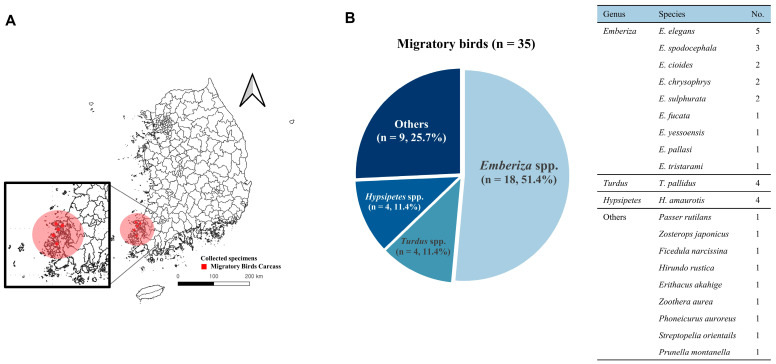
Survey areas and species distribution of migratory birds collected in South Korea. (**A**) Map showing the five coastal sampling areas in the Sinan-gun islands in the southwestern region of South Korea, where bird carcasses were collected. These areas are designated as regular monitoring sites by the Korean Ministry of Environment and serve as major stopover habitats in the East Asian–Australasian Flyway. (**B**) Species distribution of the 35 bird carcasses examined in this study. A total of 20 species were morphologically identified, including nine *Emberiza* species and 11 other species. Species identification was confirmed by field ornithologists during the necropsy.

All carcasses were collected from five coastal areas that are historically known as major habitats for migratory birds in Jeollanam-do, South Korea (Figure 1A). Among the 35 bird carcasses collected in this study, 18 birds (51.4%) were morphologically identified as *Emberiza* species: *Emberiza elegans* (*n* = 5), *E. spodocephala* (*n* = 3), *E. cioides* (*n* = 2), *E. chrysophrys* (*n* = 2), *E. sulphurata* (*n* = 2), *E. fucata* (*n* = 1), *E. yessoensis* (*n* = 1), *E. pallasi* (*n* = 1), and *E. tristarami* (*n* = 1) (Figure 1B). The remaining 17 birds were identified as *Turdus pallidus* (*n* = 4), *Hypsipetes amaurotis* (*n* = 4), *Passer rutilans* (*n* = 1), *Zosterops japonicus* (*n* = 1), *Ficedula narcissina* (*n* = 1), *Hirundo rustica* (*n* = 1), *Erithacus akahige* (*n* = 1), *Zoothera aurea* (*n* = 1), *Phoneicurus auroreus* (*n* = 1), *Streptopelia orientails* (*n* = 1), and *Prunella montanella* (*n* = 1).

**Figure 2:**
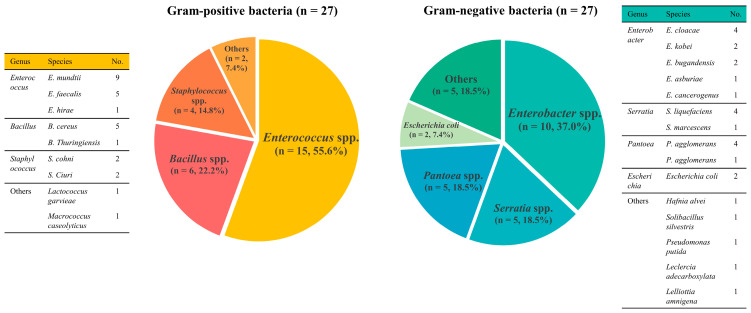
Species distribution of Gram-positive bacteria and Gram-negative bacteria isolated from migratory birds in South Korea. Bar chart summarizing the bacterial species composition of the 54 isolates from bird carcasses. Gram-positive bacteria included *Enterococcus* spp., *Bacillus* spp., *Staphylococcus* spp., and other species. Gram-negative bacteria included coliform bacteria (*Enterobacter* spp. and *Escherichia coli*), *Serratia* spp., *Pantoea* spp., and other species. The figure shows the diversity of the 24 bacterial species detected in the sampled birds and highlights the distribution patterns used for subsequent antimicrobial susceptibility analyses.

A total of 54 bacterial strains were isolated from 35 bird carcasses (Figure 2). The most frequently isolated species were *Enterococcus* spp. (*n* = 15): *E. mundtii* (*n* = 9), *E. faecalis* (*n* = 5), and *E. hirae* (*n* = 1). The other Gram-positive bacteria were identified as *Bacillus* spp. (*n* = 6), *Staphylococcus* spp. (*n* = 4), *Lactococcus garvieae* (*n* = 1), and *Macrococcus caseolyticus* (*n* = 1). Among the Gram-negative bacteria, 12 isolates belonged to the coliform group, including 10 *Enterobacter* spp. and two *E. coli*. The *Enterobacter* spp. included *Enterobacter cloacae* (*n* = 4), *E. kobei* (*n* = 2), *E. bugandensis* (*n* = 2), *E. asburiae* (*n* = 1), and *E. cancerogenus* (*n* = 1). The remaining Gram-negative bacteria were identified as *Serratia* spp. (*n* = 5), *Pantoea* spp. (*n* = 5), *Hafnia alvei* (*n* = 1), *Solibacillus silvestris* (*n* = 1), *Pseudomonas putida* (*n* = 1), *Leclercia adecarboxylata* (*n* = 1), and *Lelliottia amnigena* (*n* = 1).

### 3.2. AMR Susceptibility of Bacterial Isolates

To enhance our understanding of AMR pattern in wildlife, all bacterial isolates were subjected to AMR susceptibility test. However, the lack of standardized interpretive criteria makes it difficult to classify resistance in some species. Therefore, MIC values are provided with categorical interpretation only for *Enterococcus* spp., *Enterobacter* spp., *E. coli*, *Staphylococcus* spp., *Bacillus* spp., and *Serratia* spp. (Supplementary Table S1). Among the 54 isolates, 18 (33.3%) were identified as MDR bacteria, resistant to three or more subclasses of antimicrobial agents. These included *E. faecalis* (*n* = 5), *E. mundtii* (*n* = 3), *E. coli* (*n* = 2), *E. cloacae* (*n* = 2), *E. kobei* (*n* = 1), *E. cancerogenus* (*n* = 1), *E. bugandensis* (*n* = 1), *S. cohnii* (*n* = 1), *B. cereus* (*n* = 1), and *E. hirae* (*n* = 1).

Since *Enterococcus* spp. and coliform bacteria (*Enterobacter* spp. and *E. coli*) are key indicators used to assess fecal contamination, originating from the intestinal tracts of humans and animals, the following analysis focused on these microorganisms. For *Enterococcus* spp., the resistance rates to DAP and TIG were high at 80.0% (Table 1). The resistance rates to VAN, LZD, TYL, QUD, CIP, and SAL varied between 20.0% and 60.0%. In contrast, resistance to GEN, KAN, STR, AMP, ERY, CHL, FLO, and TET was either absent or low (0.0–13.3%). In the case of coliform bacteria, including *Enterobacter* spp. and *E. coli*, resistance rates to GEN, STR, AMP, AMC, CXI, CTA, CTZ, CEP, MER, CIP, SIS, COL, and NAL varied between 33.3% and 66.7% (Table 2). Resistance TRS, CHL, and TET was either absent or low (0.0–8.3%).

In addition to fecal indicator microorganisms, opportunistic pathogens, including *Staphylococcus* spp., *Bacillus* spp., and *Serratia* spp., for which birds can serve as infectious sources, were also analyzed. For *Staphylococcus* spp., resistance rates to CIP, ERY, CHL, and SYN ranged from 25% to 50% (Supplementary Table S2). No resistance was detected against VAN, LZD, and TET. MIC breakpoints for other antimicrobials are not available. In the case of *Bacillus* spp., resistance rates to AMP, VAN, ERY, LZD, and TET varied between 16.7% and 100.0% (Supplementary Table S3). No resistance was observed against CIP and CHL. MIC breakpoints for other antimicrobials are not available. For *Serratia* spp., resistance rates to AMC and TET were 60.0% and 20.0%, respectively (Supplementary Table S4). No resistance was observed against CTA, MER, CIP, TRS, and CHL. MIC breakpoints for other antimicrobials are not available.

## 4. Discussion

Notably, the species distribution of our necropsied carcasses does not mirror the dominant species reported in community surveys from the Sinan-gun islands. For example, bird counts on Bigeum-do and Docho-do revealed substantial populations of Barn Swallow (*Hirundo rustica*) and Eurasian Tree Sparrow (*Passer montanus*), together with coastal species such as Kentish Plover (*Charadrius alexandrines*) and Little Egret (*Egretta alba modesta*) [25]. In contrast, more than half of the carcasses in our study were *Emberiza* buntings (*E. elegans*, *E. spodocephala*, *E. cioides*, *E. chrysophrys*, *E. sulphurata*, *E. fucata*, *E. yessoensis*, *E. pallasi*, *E. tristrami*), and the remaining individuals included other small passerines such as *Turdus pallidus*, *Hypsipetes amaurotis*, *Passer rutilans*, *Zosterops japonicus*, *Ficedula narcissina*, *Hirundo rustica*, *Erithacus akahige*, *Zoothera aurea*, *Phoenicurus auroreus*, *Streptopelia orientalis* and *Prunella montanella*. This discrepancy likely arises from the differences between live-count surveys and carcass-based sampling: the former quantifies all birds present on the islands at a given time, whereas the latter depends on mortality events, drift, and recovery of dead individuals, and thus is biased towards species with higher local mortality or higher detection probability. Therefore, our organ-level bacterial data should be interpreted as being most representative of small migratory landbirds that suffered mortality during the sampling period, rather than of the entire bird community using southwestern Korean coastal habitats.

Second, the ecological context of our sampling sites helped to interpret the observed AMR patterns. The five coastal areas in Jeollanam-do from which carcasses were collected include tidal flats and island habitats that have been recognized as internationally important for migratory shorebirds and landbirds within the EAAF [18,23,24]. Long-term bird surveys in Sinan-gun and other southwestern islands have reported that *Emberiza* buntings, *Passer rutilans*, and other small passerines constitute a substantial proportion of the landbird community, exhibiting marked seasonal variations in their distribution and abundance [25]. In this study, *Emberiza* species accounted for more than half of the carcasses examined, whereas only a single *P. rutilans* individual was included, suggesting a potential bias in our sample towards species suffering higher mortality or those more likely to be found and submitted for necropsy in these coastal habitats. Nonetheless, the overlap in key bird species between our dataset and previous Sinan-gun surveys suggests that the organ-level bacterial AMR profiles reported here are likely to be broadly representative of common small migratory landbirds using southwestern Korean coastal ecosystems.

Third, temporal and ecological heterogeneity should be considered when comparing the bacterial findings of the present study with those of previous studies. Seasonal surveys in Sinan-gun and Wando-gun have documented pronounced changes in waterbird and landbird assemblages among seasons [23], and our sampling window (July–October) corresponds mainly to the late breeding and southward migration periods rather than the peak wintering season. This temporal focus, together with the small sample size, may partly explain why some classical avian bacterial pathogens such as *Salmonella* spp. or *Campylobacter* spp., which have been detected in migratory birds in other Korean studies [14,44], were not isolated from our carcasses. Instead, we observed that widely distributed commensal and environmental bacteria with opportunistic potential—*Enterococcus*, *Enterobacter*, *E. coli*, *Bacillus*, *Staphylococcus*, *Serratia*, *Pantoea* and others—were present in multiple organs, indicating ongoing exposure to fecal contamination and environmental reservoirs in the coastal agricultural landscapes where these birds forage and rest. Taken together, this ecological interpretation and the AMR profiles we report suggest that migratory birds in southwestern Korea may act as sentinels integrating signals of environmental contamination across both space (different stopover sites) and time (seasonal movements), even when the available sample size is limited.

Migrating birds have been implicated in the long-distance spread of pathogens. However, the lack of data on the microbial carriage of birds makes them promising subjects for elucidating their potential role in the transmission of antimicrobial-resistant pathogens. In this study, MDR opportunistic pathogens including *E. coli*, *E. faecalis*, *E. cloacae*, *S. cohnii*, and *B. cereus* were isolated from birds. *E. faecalis* is currently recognized as the third important nosocomial pathogen globally [45,46]. Moreover, the frequent acquisition and spread of antimicrobial resistance genes (ARGs) in *Enterococcus* have exacerbated the morbidity and mortality rates [47]. *E. cloacae* is another crucial nosocomial pathogen, associated with high morbidity and mortality among intensive care patients due to its resistance to multiple antimicrobials [48]. *S. cohnii* is an uncommon opportunistic pathogen, but it can cause bacteremia, sepsis, and urinary tract infections [49]. *B. cereus* is a spore-forming foodborne pathogen that is exhibits resistance to various environmental stresses [50]. Moreover, we reviewed previous studies to identify additional or unexpected bacterial species; however, no novel species were detected in this study. Nevertheless, even commonly reported bacteria are rarely found in the internal organs of migratory birds, and such information remains limited. For a more comprehensive study on bacterial diversity, further research should include additional sampling seasons and geographic location to explore novel pathogens and AMR patterns.

Although migratory birds are increasingly recognized as reservoirs and disseminators of AMR, research on this topic in South Korea is limited. In the 2010s, two studies addressed this issue. Oh et al. [44] identified plasmid-mediated quinolone resistance (PMQR) genes, such as *qnrS* and *aac(6′)-Ib-cr*, in *E. coli* isolated from various bird species, suggesting the potential for long-distance spread of PMQR genes through bird migration [44]. Another study investigated the prevalence and AMR profile of *Campylobacter* spp. isolates from migratory birds and found low resistance rates to CIP (3.0%) and TET (1.8%) [14]. However, both studies were limited to specific bacterial species and sampling periods around 2009–2010. Since then, no reports on AMR among bacterial isolates from migratory birds in South Korea has been published. This prolonged research gap contrasts with the expanding surveillance efforts in livestock and humans, leaving the ecological role of birds in AMR transmission largely unexplored in South Korea to date.

In this study, we identified MDR bacterial isolates from migratory birds in South Korea. The detection of resistance to VAN, LZD, TIG, and DAP in *Enterococcus* isolates raises serious public health concerns. Although VAN has been used as a crucial antimicrobial for treating MDR *Enterococcus*, the emergence of VAN resistance in these strains has created a therapeutic dilemma for clinicians [51]. Currently, only a few last-resort antimicrobials, including LZD, DAP, and TIG, are available for treating VRE [52]. Notably, we report for the first time that two VRE isolates (one *E. mundtii* and one *E. faecalis*) from migratory birds in South Korea exhibited resistance to LZD, DAP, and TIG. Such concurrent resistance to VAN and last-resort antimicrobials in *Enterococcus* suggest the environmental spread of AMR originated from hospitals and agricultural sites [53,54]. In addition, coliform bacterial isolates exhibited the resistance to CIAs, including third- and fourth-generation cephalosporins, carbapenems, and colistin. Notably, our two *E. coli* isolates exhibited concurrent carbapenem-colistin resistance. Such AMR profiles are concerning, as these antimicrobials are last-resort agents for treating MDR Gram-negative bacterial infections in humans [55]. Although *Enterococcus* and *E. coli* are commensal bacteria, they are commonly implicated in infections in human and animals [56,57]. This necessitates the use of antimicrobials and raise public health concerns due to the spread of MDR *Enterococcus* and *E. coli* into wildlife.

Intrinsic resistance, unlike acquired resistance, arises from chromosomally encoded mechanisms that naturally exist in bacterial species and do not rely on horizontal gene transfer. For example, *Enterococcus* spp. exhibit intrinsic resistance to clindamycin and QUD because of the presence of endogenous *lsa* genes, which encode ATP-binding efflux pumps [58]. Similarly, *Enterobacter* spp. have inducible chromosomal *ampC* β-lactamase genes that confer intrinsic resistance to ampicillin and first-generation cephalosporins [59]. These intrinsic mechanisms complicate the interpretation of multidrug resistance profiles, particularly in isolates from environments with limited antibiotic exposure, such as migratory birds. Therefore, distinguishing intrinsic from acquired resistance is essential for accurate ecological risk assessment and understanding the baseline resistome of wildlife populations. Future genomic investigations should include the characteristics of these intrinsic determinants. Nevertheless, AMR patterns observed in our *Enterococcus* isolates from the internal organs of migratory birds, rather than from environmental or fecal sources, are rarely reported and provide scientific novelty.

## 5. Conclusions

This study provides the first organ-level evidence that migratory birds in South Korea harbor diverse MDR bacteria, including VRE and carbapenem- and colistin-resistant *E. coli*. These findings highlight the potential role of migratory birds as ecological sentinels and reservoirs contributing to the environmental dissemination of high-risk AMR. Continued One Health-based wildlife surveillance is essential to better characterize AMR transmission pathways and assess associated public health risks. However, it should be recognized that the present study has some limitations. First, the small sample size limits the representativeness of our findings. Collecting fresh migratory bird carcasses suitable for necropsy and organ sampling is challenging for the following reasons—(i) the mortality events of migratory birds are unpredictable, (ii) carcasses must be collected rapidly before decomposition to avoid microbiological alterations, (iii) weather and predation can rapidly degrade carcasses. Therefore, although we cannot calculate the prevalence or characterize bacterial diversity at the population level, we can identify the presence of clinically significant AMR phenotypes in migratory birds, which can be achieved with a modest sample size. Second, the potential contamination by environmental bacteria cannot be excluded. Third, the antimicrobial susceptibility test based solely on the broth microdilution method, which does not reveal the genetic mechanisms of resistance. Since this study represents a preliminary investigation, future research with an expanded sample size, broader species coverage, and enhanced bacteriological profiling is required. Despite these limitations, we provide valuable baseline data for incorporating wildlife surveillance into the national monitoring frameworks, which aligns with the scope of veterinary sciences.

## Figures and Tables

**Table 1:** Results obtained testing 15 *Enterococcus* spp. isolates versus 16 antimicrobials with the broth microdilution method test.

Antimicrobials		Susceptible		Intermediate		Resistant	
Class	Molecules ^a^	Number of Isolates	%	Number of Isolates	%	Number of Isolates	%
Aminoglycosides	GEN	15	100.0	0	0.0	0	0.0
	KAN	15	100.0	0	0.0	0	0.0
	STR	15	100.0	0	0.0	0	0.0
Aminopenicillin	AMP	13	86.7	0	0.0	2	13.3
Fluoroquinolone	CIP	4	26.7	6	40.0	5	33.3
Glycopeptide	VAN	12	80.0	0	0.0	3	20.0
Glycylcyclines	TIG	2	20.0	0	0.0	13	80.0
Lipopeptides	DAP	1	6.7	2	13.3	12	80.0
Macrolides	ERY	8	53.3	5	33.3	2	13.3
	TYL	12	80.0	0	0.0	3	20.0
Oxazolidinones	LZD	1	6.7	11	73.3	3	20.0
Phenicols	CHL	2	13.3	11	73.3	2	13.3
	FLO	14	93.3	0	0.0	1	6.7
Streptogramins	QUD	5	33.3	1	6.7	9	60.0
Tetracyclines	TET	14	93.3	0	0.0	1	6.7
Others	SAL	12	80.0	0	0.0	3	20.0

^a^ GEN, gentamicin; KAN, kanamycin; STR, streptomycin; AMP, ampicillin; CIP, ciprofloxacin; VAN, vancomycin; TIG, tigecycline; DAP, daptomycin; ERY, erythromycin; TYL, tylosin; LZD, linezolid; CHL, chloramphenicol; FLO, florfenicol; QUD, quinupristin/dalfopristin; TET, tetracycline; SAL, salinomycin.

**Table 2:** Results obtained testing 12 coliform bacterial isolates versus 16 antimicrobials with the broth microdilution method test.

Antimicrobials		Susceptible		Intermediate		Resistant	
Class	Molecules ^a^	Number of Isolates	%	Number of Isolates	%	Number of Isolates	%
Aminoglycosides	GEN	8	66.7	0	0.0	4	33.3
	STR	6	50.0	0	0.0	6	50.0
Aminopenicillin	AMP	5	41.7	2	16.7	5	41.7
β-lactam/β-lactmase inhibitor	AMC	2	16.7	4	33.3	6	50.0
Cephamycin	CXI	0	0.0	4	33.3	8	66.7
Cephalosporin III	CTA	7	58.3	0	0.0	5	41.7
	CTZ	7	58.3	0	0.0	5	41.7
Cephalosporin IV	CEP	7	58.3	0	0.0	5	41.7
Carbapenem	MER	7	58.3	0	0.0	5	41.7
Fluoroquinolone	CIP	7	58.3	0	0.0	5	41.7
Folate pathway inhibitors	TRS	11	91.7	0	0.0	1	8.3
	SIS	5	41.7	0	0.0	7	58.3
Phenicols	CHL	7	58.3	5	41.7	0	0.0
Polymyxins	COL	0	0.0	6	50.0	6	50.0
Quinolone	NAL	7	58.3	0	0.0	5	41.7
Tetracyclines	TET	9	75.0	2	16.7	1	8.3

^a^ GEN, gentamicin; STR, streptomycin; AMP, ampicillin; AMC, amoxicillin/clavulanic acid; CXI, cefoxitin; CTA, cefotaxime; CTZ, ceftazidime; CEP, cefepime; MER, meropenem; CIP, ciprofloxacin; TRS, trimethoprim/sulfamethoxazole; SIS, sulfisoxazole; CHL, chloramphenicol; COL, colistin; NAL, nalidixic acid; TET, tetracycline.

## Data Availability

The original contributions presented in this study are included in the article/Supplementary Material. Further inquiries can be directed to the corresponding authors.

## Supplementary Materials

The following supporting information can be downloaded at https://www.mdpi.com/article/10.3390/vetsci12121157/s1. Table S1: Antimicrobial resistance profiles of 54 bacterial isolates from 35 migratory birds; Table S2: MICs of 16 antimicrobial agents against 4 *Staphylococcus* spp. isolates; Table S3: MICs of 16 antimicrobial agents against 6 *Bacillus* spp. isolates; Table S4: MICs of 16 antimicrobial agents against 5 *Serratia* spp. isolates.
