# Supplementary material for: Ecological Diversity of Migratory Birds and Their Associated Bacterial Species in South Korea: A Preliminary Study Including Antimicrobial Resistance Profiles
Source: Vet Sci. 2025 Dec 4;12(12):1157. doi: 10.3390/vetsci12121157 (PMC12737476; doi:10.3390/vetsci12121157)
Supplement: Supplementary file 1 [file vetsci-12-01157-s001.zip › vetsci-3975300-supplementary.pdf]

Table S1. Antimicrobial resistance profiles of 54 bacterial isolates from 35 migratory birds.

No.	Bird species	Organs	Bacterial isolates[a]	Antibiotic resistance profiles[b]
1	*Emberiza spodocephala*	Bursa of Fabricius	*Enterobacter asburiae*	None[c]
			Pantoea agglomerans	Not interpretable[d]
			Bacillus cereus	VAN, AMP, ERY, LZD
		Spleen	*Enterobacter cloacae*	None
2	*Emberiza chrysophrys*	Liver	*Enterococcus mundtii*	VAN, DAP, ERY, TYL, SAL, LZD, QUD, TIG
			Hafnia alvei	Not interpretable
3	*Passer rutilans*	Liver	*Enterococcus mundtii*	CIP, DAP, QUD, TIG
			Staphylococcus cohnii	CIP, CHL, ERY, QUD
4	*Emberiza elegans*	Liver	*Bacillus thuringiensis*	TET, AMP
5	*Emberiza elegans*	Liver	*Enterococcus mundtii*	DAP
			Enterobacter kobei	None
			Bacillus cereus	AMP
6	*Emberiza sulphurata*	Liver	*Enterococcus mundtii*	DAP, TIG
7	*Emberiza cioides*	Liver	*Enterococcus mundtii*	DAP
8	*Turdus pallidus*	Liver	*Enterococcus mundtii*	DAP, TIG
			Enterobacter cloacae	None
9	*Emberiza elegans*	Heart	*Staphylococcus sciuri*	None
			Enterococcus hirae	TET, DAP, CHL, QUD, TIG
		Liver	*Enterococcus mundtii*	TIG
10	*Emberiza elegans*	Spleen	*Solibacillus silvestris*	Not interpretable
11	*Emberiza fucata*	Liver	No bacteria grown	
12	*Emberiza chrysophrys*	Liver	*Enterococcus mundtii*	VAN, AMP, DAP, CHL, ERY, FLO, TYL, SAL, LZD, QUD
		Heart	*Pseudomonas putida*	Not interpretable
			Staphylococcus cohnii	QUD
13	*Emberiza sulphurata*	Liver	*Staphylococcus sciuri*	None
14	*Emberiza cioides*	Liver	*Enterococcus mundtii*	DAP, TIG
15	*Turdus pallidus*	Liver	*Escherichia coli*	CEP, CXI, CIP, STR, GEN, CTZ, NAL, CTA, MER, COL
			Enterobacter kobei	AMC, CEP, CXI, AMP, CIP, GEN, CTZ, NAL, CTA, MER, COL
16	*Zosterops japonicus*	Liver	*Enterobacter cloacae*	AMC, CXI, AMP, TET, COL

17	*Ficedula narcissina*	Liver	*Lactococcus garvieae*	Not interpretable
			Enterococcus faecalis	VAN, AMP, DAP, ERY, TYL, SAL, LZD, QUD, TIG
18	*Hirundo rustica*	Liver	*Serratia marcescens*	AMC, TET
19	*Erithacus akahige*	Liver	*Macrococcus caseolyticus*	Not interpretable
			Leclercia adecarboxylata	Not interpretable
			Serratia liquefaciens	AMC
			Escherichia coli	CEP, CXI, QUD, AMP, CIP, STR, GEN, CTZ, NAL, CTA, MER, COL
20	*Emberiza yessoensis*	Liver	*Serratia liquefaciens*	AMC
			Bacillus cereus	AMP
21	*Emberiza spodocephala*	Liver	*Serratia liquefaciens*	None
		Bursa of Fabricius	*Pantoea agglomerans*	Not interpretable
22	*Hypsipetes amaurotis*	Bursa of Fabricius	*Pantoea agglomerans*	Not interpretable
23	*Emberiza pallasi*	Spleen	*Pantoea agglomerans*	Not interpretable
24	*Hypsipetes amaurotis*	Liver	No bacteria grown	
25	*Zoothera aurea*	Bursa of Fabricius	No bacteria grown	
		Spleen	No bacteria grown	
26	*Phoenicurus auroreus*	Liver	*Enterococcus faecalis*	CIP, DAP, QUD
27	*Turdus pallidus*	Liver	*Serratia liquefaciens*	None
28	*Emberiza tristrami*	Liver	*Lelliottia amnigena*	Not interpretable
29	*Emberiza spodocephala*	Liver	*Enterococcus faecalis*	CIP, DAP, QUD, TIG
			Bacillus cereus	LZD, AMP
30	*Streptopelia orientails*	Liver	*Enterobacter cancerogenus*	AMC, CEP, CXI, CIP, STR, CTZ, SIS, NAL, CTA, MER, COL
31	*Turdus pallidus*	Liver	*Enterobacter bugandensis*	AMC, CXI
		Bursa of Fabricius	*Enterobacter cloacae*	AMC, CXI, AMP, STR, SIS
32	*Hypsipetes amaurotis*	Bursa of Fabricius	*Enterococcus faecalis*	CIP, QUD, TIG

33	*Prunella montanella*	Spleen	*Enterobacter bugandensis*	AMC, CEP, CXI, AMP, CIP, STR, GEN, CTZ, SIS, NAL, CTA, MER, COL
			Pantoea agglomerans	Not interpretable
34	*Emberiza elegans*	Liver	*Enterococcus faecalis*	CIP, QUD, TIG
35	*Hypsipetes amaurotis*	Liver	*Bacillus cereus*	AMP

[a] Bacteria highlighted in gray color indicate the multidrug-resistant strains resistant to three or more subclass of antimicrobial agents.

[b] TET, tetracycline; VAN, vancomycin; CIP, ciprofloxacin; AMP, ampicillin; DAP, daptomycin; CHL, chloramphenicol; ERY, erythromycin; TYL, tylosin; SAL, salinomycin; LZD, linezolid; QUD, quinupristin/dalfopristin; TIG, tigecycline; AMC, amoxicillin/clavulanic acid; CEP, cefepime; CXI, cefoxitin; TRS, trimethoprim/sulfamethoxazole; STR, streptomycin; GEN, gentamicin; CTZ, ceftazidime; NAL, nalidixic acid; CTA, cefotaxime; MER, meropenem; COL, colistin.

[C] This bacterial isolate was susceptible to all antimicrobials used in this study.

[d] Since no established interpretive criteria exist for bacterial species-antibiotic combinations, it was not scientifically appropriate to assign resistance categories. To maintain methodological rigor and data transparency, these isolates were reported as “not interpretable” rather than being excluded or arbitrarily categorized.

Table S2. MICs of 16 antimicrobial agents against 4 *Staphylococcus* spp. isolates

Antimicrobials		MIC Values (µg/ml)[b, c]														Resistant (%)
Class	Molecules[a]	0.12	0.25	0.5	1	2	4	8	16	32	64	128	256	512	1024	
Aminoglycosides	GEN										4					
	KAN										4					
	STR										4					
Aminopenicillin	AMP			3						1						
Fluoroquinolone	CIP	1		2						1						25.0
Glycopeptide	VAN				4											0.0
Glycylcyclines	TIG	1		3												
Lipopeptides	DAP			0	1	2										
Macrolides	ERY			3								1				25.0
	TYL															
Oxazolidinones	LZD			1		3										0.0
Phenicols	CHL						1	2			1					25.0
	FLO															
Streptogramins	QUD					2	1	1								50.0
Tetracyclines	TET				4											0.0
Others	SAL															

[a] GEN, gentamicin; KAN, kanamycin; STR, streptomycin; AMP, ampicillin; CIP, ciprofloxacin; VAN, vancomycin; TIG, tigecycline; DAP, daptomycin; ERY, erythromycin; TYL, tylosin; LZD, linezolid; CHL, chloramphenicol; FLO, florfenicol; QUD, quinupristin/dalfopristin; TET, tetracycline; SAL, salinomycin.

[b] White cells indicate the dilution range tested. Vertical lines describe the susceptible and resistant breakpoints recommended by CLSI, NARMS, and EUCAST.

[c] No established interpretive criteria exist for STR, AMP, TIG, DAP, TYL, FLO, and SAL.

Table S3. MICs of 16 antimicrobial agents against 6 *Bacillus* spp. isolates

Antimicrobials		MIC Values (µg/ml)[b, c]														Resistant (%)
Class	Molecules[a]	0.12	0.25	0.5	1	2	4	8	16	32	64	128	256	512	1024	
Aminoglycosides	GEN										6					
	KAN										6					
	STR										6					
Aminopenicillin	AMP								3	3						100.0
Fluoroquinolone	CIP	5				1										0.0
Glycopeptide	VAN				5						1					16.7
Glycylcyclines	TIG		3	3												
Lipopeptides	DAP				2			2	1		1					
Macrolides	ERY			4		1					1					16.7
	TYL			4			1					1				
Oxazolidinones	LZD		1	1	2	1				1						33.3
Phenicols	CHL				1		4	1								0.0
	FLO				5		1									
Streptogramins	QUD			1	2	1			1		1					
Tetracyclines	TET				4		1				1					16.7
Others	SAL				5						1					

[a] GEN, gentamicin; KAN, kanamycin; STR, streptomycin; AMP, ampicillin; CIP, ciprofloxacin; VAN, vancomycin; TIG, tigecycline; DAP, daptomycin; ERY, erythromycin; TYL, tylosin; LZD, linezolid; CHL, chloramphenicol; FLO, florfenicol; QUD, quinupristin/dalfopristin; TET, tetracycline; SAL, salinomycin.

[b] White cells indicate the dilution range tested. Vertical lines describe the susceptible and resistant breakpoints recommended by CLSI, NARMS, and EUCAST.

[c] No established interpretive criteria exist for KAN, STR, AMP, TIG, DAP, TYL, FLO, QUD, and SAL.

Table S4. MICs of 16 antimicrobial agents against 5 *Serratia* spp. isolates

Antimicrobials		MIC Values (µg/ml)[a, b]													Resistant (%)
Class	Molecules[a]	0.12	0.25	0.5	1	2	4	8	16	32	64	128	256	512	
Aminoglycosides	GEN			5											
	STR							5							
Aminopenicillin	AMP							2	2			1			
β-lactam/β-lactmase inhibitor combination	AMC							2		1	2				60.0
Cephamycin	CXI			2				2	2	1					
Cephalosporin III	CTA		5												0.0
	CTZ			5											
Cephalosporin IV	CEP	5													
Carbapenem	MER	5													0.0
Fluoroquinolone	CIP	4	1												0.0
Folate pathway	TRS	2	3												0.0
inhibitors	SIS										1			4	
Phenicols	CHL						2	2	1						0.0
Polymyxins	COL								1	4					
Quinolone	NAL				5										
Tetracyclines	TET					1	2	1		1					20.0

[a] GEN, gentamicin; STR, streptomycin; AMP, ampicillin; AMC, amoxicillin/clavulanic acid; CXI, cefoxitin; CTA, cefotaxime; CTZ, ceftazidime; CEP, cefepime; MER, meropenem; CIP, ciprofloxacin; TRS, trimethoprim/sulfamethoxazole; SIS, sulfisoxazole; CHL, chloramphenicol; COL, colistin; NAL, nalidixic acid; TET, tetracycline.

[b] White cells indicate the dilution range tested. Vertical lines describe the susceptible and resistant breakpoints recommended by CLSI, NARMS, and EUCAST.

[c] No established interpretive criteria exist for GEN, STR, AMP, CXI, CTZ, CEP, SIS, COL, and NAL.
